# Fifth District Dental Society of the State of New York

**Published:** 1884-05

**Authors:** 


### FIFTH DISTRICT DENTAL SOCIETY OF THE STATE OF NEW YORK.

The sixteenth annual meeting was held at Utica, April 8th and 9th, 1884. President, Dr. G. V. N. Relyea, Oswego; Secretary, Dr. G. L. Curtis, Syracuse.

The first session was mainly occupied with routine business, and the reading of the annual address by the president.

At the evening session a paper was read by Dr. S. B. Palmer, upon "Dental Hygiene; the best Varieties of Food to Develop and Sustain Tooth Structure"; (See page 237). At its conclusion the members separated into parties of four or five, and discussed the subject, thus giving an opportunity to obtain the views of all, and the substance of the whole was subsequently reported to the meeting by the chairman of the several sections. The general tone of the remarks was in favor of the coarser foods, the use of phosphates, and the promotion of exercise of the teeth and jaws by a resistant diet.

At the morning session of Wednesday Dr. A. Retter read a paper upon "Dental Mechanism." (See page 233).

*Dr. Emmons*—Said that he had been conducting a series of experiments with rubber of different manufacturers to determine the comparative strength. The first specimen broke under a strain of two pounds; the second at six pounds, four ounces; the third at nine pounds, seven ounces; the fourth at thirteen pounds, six ounces; the fifth at twenty-four pounds, three ounces; the sixth at twenty-seven pounds, two ounces; showing a wide difference in the strength of different manufactures of rubber.

*Dr. Relyea*—Said that he had sometimes obtained an impression cup for difficult cases by modeling wax over a cast drawn from a wax impression, and covering it with several coats of shellac.

*Dr. Emmons*—Had moulded rubber impression cups over such a plaster-cast.

*Dr. Retter*—Gave his experience in the use of bromide of ethyl, as an anæsthetic, and subsequently submitted to its administration at the hands of Dr. G. L. Curtis. The anæsthesia was very profound, was accompanied by no signs of nausea, and the return to entire consciousness was easy and natural, the period of anæsthesia being less than two minutes.

An invitation was received to hold the semi-annual meeting in conjunction with the Sixth District Society, at Syracuse, on the second Tuesday in October.

The following officers were elected :

President—Dr. C. E. Cherry, Syracuse.
Vice President—Dr. C. C. Smith, Ilion.
Recording Secretary—Dr. G. L. Curtis, Syracuse.
Corresponding Secretary—Dr. B. T. Mason, Phœnix.
Treasurer—Dr. J. C. House, Lowville.
Librarian—Dr. A. N. Priest, Utica.

